# Dietary Encapsulation of a Novel *Lysinibacillus* sp. PWR01 Probiotic Modulates Growth, Antioxidant, Immune Gene Expression, and Gut Health in Nile tilapia (*Oreochromis niloticus*) Against *Aeromonas hydrophila* Infection

**DOI:** 10.3390/antiox15030373

**Published:** 2026-03-16

**Authors:** Nguyen Vu Linh, Luu Tang Phuc Khang, Suwanna Wisetkaeo, Nguyen Dinh-Hung, Papungkorn Sangsawad, Waraphorn Sihamok, Orathai Dangsawat, Kritsada Phetduang, Phatthanaphong Therdtatha, Mintra Seel-audom, Patima Permpoonpattana

**Affiliations:** 1 Department of Animal and Aquatic Sciences, Faculty of Agriculture, Chiang Mai University, Chiang Mai 50200, Thailand; linhvu.n@cmu.ac.th (N.V.L.); khang_luu@cmu.ac.th (L.T.P.K.); suwanna_w@cmu.ac.th (S.W.); mintra.s@cmu.ac.th (M.S.-a.); 2 Aquaculture Pathology Laboratory, School of Animal & Comparative Biomedical Sciences, The University of Arizona, 1117 E Lowell St., Tucson, AZ 85721, USA; dinhhung@arizona.edu; 3 School of Animal Technology and Innovation, Institute of Agricultural Technology, Suranaree University of Technology, Nakhon Ratchasima 30000, Thailand; papungkorn@sut.ac.th; 4 Department of Agricultural Science and Technology, Faculty of Innovative Agriculture, Fisheries and Food, Prince of Songkla University, Surat Thani Campus, Surat Thani 84000, Thailand; 6540620103@psu.ac.th; 5 Scientific Laboratory and Equipment Center, Office of Surat Thani Campus, Prince of Songkla University, Surat Thani Campus, Surat Thani 84000, Thailand; orathai.d@psu.ac.th; 6 Specialized Research in Microbiome and Metabolome for Health Laboratory, Division of Biotechnology, Faculty of Agro-Industry, Chiang Mai University, Chiang Mai 50200, Thailand; kritsada_phetd@cmu.ac.th (K.P.); phatthanaphong.th@cmu.ac.th (P.T.)

**Keywords:** antioxidant activity, disease resistance, gut bacteria, probiotic supplementation, sustainable aquaculture

## Abstract

Encapsulated probiotics, which are promising approaches for improving aquaculture species’ performance and health, have incompletely characterized dose-dependent physiological and immunological effects. This study represents the first report evaluating the probiotic efficacy of a novel encapsulated *Lysinibacillus* sp. PWR01, originally isolated from rubber latex nodules, in Nile tilapia (*Oreochromis niloticus*). A total of 300 Nile tilapia (10.80 ± 0.07 g) was allocated to four experimental groups receiving 0, 10^4^, 10^5^, and 10^6^ CFU/g of encapsulated *Lysinibacillus* sp. PWR01 in their diet. At 8 weeks of the feeding trial, growth performance, feed utilization, serum antioxidant status, intestinal bacterial counts and immune-related gene expression were analyzed. Results demonstrated that fish fed 10^6^ CFU/g achieved a final weight of 51.48 g, representing a 9.88% increase compared with the control (46.85 g), while weight gain (WG) improved by 12.82% and specific growth rate (SGR) by 6.34%. Feed conversion ratio (FCR) was reduced by up to 18.42% at 10^5^ CFU/g relative to the control. Encapsulated groups enhanced serum superoxide dismutase activity without altering malondialdehyde levels, increased total intestinal bacterial and lactic acid bacteria counts, and selectively upregulated *TLR2* and *MHC II* mRNA transcript levels. Histological analysis further revealed increased intestinal villus height and width in encapsulated-fed groups, while liver architecture remained normal across treatments. Multivariate analyses demonstrated strong positive associations among growth performance, gut microbiota enrichment, and immune gene expression. Resistance to *Aeromonas hydrophila* infection was significantly improved at higher dietary doses, with post-challenge survival reaching 61.67–75% in supplemented groups versus 45.00% in controls. These findings demonstrate that latex-derived *Lysinibacillus* sp. PWR01 acts as a strain-specific immunomodulatory probiotic that enhances growth, antioxidant capacity, microbial community balance, and disease resistance to Nile tilapia. Future studies should investigate the role of the Nrf2 antioxidant pathway, clarify TLR2-mediated immune signaling, and assess gut microbiota–immune system interactions within commercial-scale production systems.

## 1. Introduction

Aquaculture is the world’s fastest-growing food production sector, now providing over half of all fish for human consumption [1]. Nile tilapia (*Oreochromis niloticus*) is one of the most widely farmed freshwater fish globally, valued for its rapid growth and hardiness, and its production has reached record highs in recent years [2,3,4]. Intensive farming practices needed to meet rising demand often lead to high stocking densities and degraded water quality, which increase fish stress and susceptibility to diseases [5,6]. Bacterial outbreaks in tilapia culture cause severe economic losses; for example, *Aeromonas hydrophila* (*A. hydrophila*), the causative agent of motile *Aeromonas* septicemia, is a prevalent pathogen that can induce high mortality in tilapia populations [7,8]. Therefore, ensuring fish health and disease resistance in such intensive systems has become a paramount challenge in aquaculture.

Historically, disease control in aquaculture has relied on antibiotics. However, the overuse of antibiotics has led to the emergence of antibiotic-resistant bacteria and residual drug accumulation in the environment [9,10]. These issues pose risks to animal and human health, prompting many countries to restrict antibiotic use in farmed fish. In response, there is growing interest in sustainable, eco-friendly disease management strategies. Probiotics have emerged as a viable alternative to antibiotics in aquaculture, offering a means to prevent infections through modulation of the host microbiota and immunity rather than chemical therapeutics [11,12,13]. The use of probiotics in fish farming has expanded significantly in the past decade [14], reflecting their promise in improving fish health and reducing dependence on antimicrobial drugs.

Probiotics are defined as viable beneficial microorganisms that, when delivered to the host in sufficient quantities, confer measurable health benefits [15,16]. In aquaculture, dietary probiotic supplementation has been extensively reported to enhance growth performance, feed utilization efficiency, and immunological responses of fish [16,17,18]. Previous studies have shown that probiotic supplementation can increase weight gain (WG), improve feed conversion ratio (FCR), and stimulate the fish immune system, thereby improving survival rate against bacterial infections [19,20,21,22]. Among the diverse available candidates for aquaculture application, *Bacillus* species are especially popular in aquaculture due to their spore-forming ability, which confers high stability in feed and the gastrointestinal tract [23]. Numerous studies on Nile tilapia have demonstrated the benefits of *Bacillus*-based probiotics. Dietary inclusion of *Bacillus* (e.g., *B. subtilis*, *B. licheniformis* or their combinations) has been shown to significantly promote growth performance, enhance innate immune parameters, and elevate resistance to bacterial pathogens [24,25,26]. These beneficial outcomes are mechanistically attributed to improved nutrient digestibility, favorable modulation of gut microbiota composition toward health-promoting communities, and production of bioactive compounds that inhibit pathogens [27,28]. As a consequence, *Bacillus*-based probiotics have firmly become an effective and widely adopted feed additive in tilapia culture to sustain productivity and host health. Parallel to this well-established literature, contemporary research is increasingly directing attention toward novel bacterial genera with underexplored probiotic potential. *Lysinibacillus*, a spore-forming genus phylogenetically closed to *Bacillus* has emerged as a candidate of particular interest. Recent investigations have demonstrated that select *Lysinibacillus* strains exhibit broad-spectrum antibacterial activity against major aquatic pathogens, including *A. hydrophila*, *Vibrio* spp., and *Streptococcus* spp. [29,30,31], suggesting functional diversity same as typically documented for conventional *Bacillus* probiotics. In addition, *Lysinibacillus* species have shown promising probiotic properties in preliminary studies. Yao, et al. [31] isolated a *Lysinibacillus* strain from the gut of yellow croaker (*Larimichthys crocea*) that exhibited strong antimicrobial activity against several fish pathogens, including *A. hydrophila*. These findings indicate that *Lysinibacillus* could serve as a beneficial probiotic in aquaculture. Notably, *Lysinibacillus* shares the advantages of spore formation and environmental resilience with *Bacillus* [32], but its use in fish diets has not been as extensively studied. To date, most evidence for *Lysinibacillus* efficacy comes from in vitro assays or trials in species like zebrafish (*Danio rerio*) [30], whiteleg shrimp (*Litopenaeus vannamei*) [29] or common carp (*Cyprinus carpio*) [33] rather than in major culture species like tilapia. Those results underscore the probiotic potential of *Lysinibacillus*; however, there remains a clear knowledge gap regarding its in vivo effects in economically important fish like tilapia, especially under practical farming conditions.

Another major determinant of probiotic efficacy in aquafeeds is delivery performance and viability at the intestinal target site. To exert beneficial effects, probiotic cells must successfully withstand feed processing, storage, immersion/leaching in water, and the acidic and bile-rich conditions of the gastrointestinal tract [34,35]. Even in spore-forming bacteria, viability losses and suboptimal intestinal colonization can occur, which may reduce functional dosing and compromise consistency of outcomes, particularly in intensive systems where stressors are frequent [36]. Although *Lysinibacillus* species possess endospores, there is limited empirical evidence regarding their survival efficiency in tilapia gastrointestinal conditions when administered in non-encapsulated form. Potential reductions in viable cell delivery to the intestine may therefore constrain probiotic efficacy and justify the exploration of protective delivery systems [36]. To address these limitations, encapsulation technologies (e.g., alginate-based microcapsules and related biopolymer matrices) have increasingly been adopted to protect probiotics from processing stress and gastrointestinal degradation, reduce premature leaching in water, and enable controlled release within the intestine [37,38]. Recent aquaculture studies have demonstrated that encapsulation can significantly enhance probiotic survival during feed processing and improve intestinal recovery rates compared with free-cell formulations [39,40,41], leading to more consistent physiological and immunological outcomes.

Critically, no peer-reviewed investigation has thus far examined the efficacy of an encapsulated *Lysinibacillus* strain in Nile tilapia subjected to *A. hydrophila* challenge, leaving a critical translational gap in both delivery strategy and host–pathogen context. Although preliminary trials in model species suggest probiotic potential, direct evidence in Nile tilapia remains lacking. This gap limits assessment of whether *Lysinibacillus* can provide comparable or complementary benefits to established *Bacillus*-based probiotics in tilapia aquaculture. Therefore, the current study aims to investigate dietary encapsulated *Lysinibacillus* sp. PWR01 in Nile tilapia, with emphasis on growth performance, antioxidant status, intestinal bacterial counts, immune-related gene expression, and resistance to *A. hydrophila* infection. By integrating an emerging probiotic with a protective delivery technology and evaluating its efficacy under pathogen challenge in a major aquaculture species, this study addresses a clearly defined research gap and contributes to the translational development of *Lysinibacillus*-based probiotics for tilapia culture.

## 2. Materials and Methods

### 2.1. Bacterial Culture and Experimental Fish

*Lysinibacillus* sp. PWR01 was isolated, characterized, and identified from our previous study [37]. The *16S rRNA* sequencing data has been uploaded to the National Center for Biotechnology Information (NCBI) database under accession number PX415241.1. The strain was originally isolated from rubber latex nodules collected from a rubber plantation in Surat Thani Province, Thailand. The isolate was cultured on nutrient agar (NA; HiMedia, Thane, India).

The bacterial pathogen *A. hydrophila* used for the challenge experiment was isolated from diseased fish during an outbreak at a local aquaculture farm and was obtained from the Centex Shrimp Center, Faculty of Science, Mahidol University, Thailand.

A total of 300 one-month-old Nile tilapia were obtained from a local fish farm in Chiang Mai Province, Thailand. At the start of the experiment, fish had a mean body weight of 10.80 ± 0.07 g. Fish were acclimated under laboratory conditions prior to the feeding trial and challenge test. Before the experiment commenced, 10 randomly selected fish were screened to confirm the absence of bacterial and viral infections.

### 2.2. Diet Preparation

The experimental diets followed a protocol from a previous study [38]. Briefly, polysaccharides were extracted from dried *Gracilaria fisheri* seaweed collected from Thung Sai Chai, Chaiya District, Surat Thani Province, Thailand. Dried seaweed (20 g) was soaked in a 6% (*w*/*v*) sodium hydroxide (NaOH) solution at 28 °C for 5 h. Rinsed with distilled water, the seaweed was cut into 1.0–1.5 cm segments and hot-water extracted. Distilled water was added to the mixture, and the pH was adjusted to 6.2–6.8. The mixture was boiled for 1.5 h, stirred occasionally, and then filtered through two layers of fine cloth. The filtrate was frozen at −20 °C for 24 h, dried in a hot-air oven at 60 °C until constant weight, ground into a fine powder, and stored at room temperature for feed formulation.

**Figure 1:**
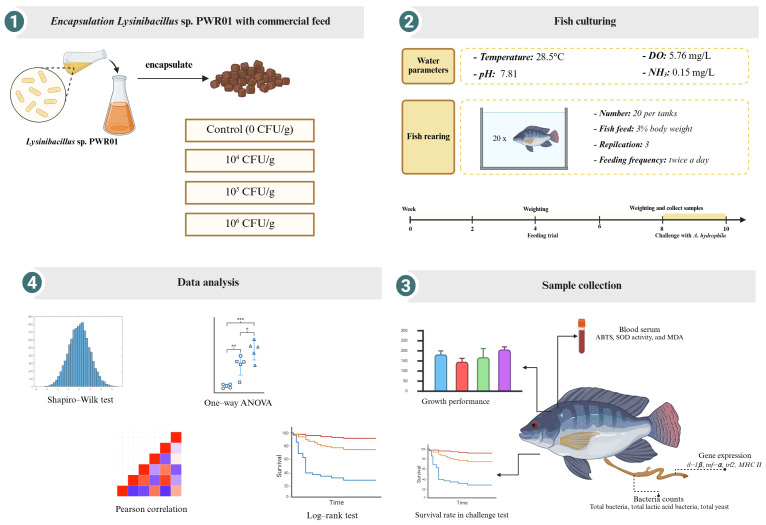
Schematic overview of the experimental design. Asterisks denote the level of statistical significance, where * indicates *p* < 0.05, ** indicates *p* < 0.01, and *** indicates *p* < 0.001.

Probiotic diets were formulated by incorporating *Lysinibacillus* sp. PWR01 spores. Spore production was accomplished through nutrient depletion in Difco Sporulation Medium. Subsequently, the purified spores were enumerated and purified according to the methodology outlined by Goncalves, et al. [42]. The purified spores were incorporated into the experimental diets at concentrations of 0 (control), 10^4^, 10^5^, and 1 × 10^6^ CFU/g of feed (Figure 1).

For encapsulation, purified spores were suspended in a sterile sodium alginate at a final concentration of 2% (*w*/*v*). Encapsulation was performed by the dropwise addition of this mixture into a 0.45 M CaCl_2_ solution under continuous gentle stirring, allowing bead solidification to proceed for 45 min at room temperature. The resultant gel beads, with a mean particle diameter of approximate [4–5 mm], were collected through filtration using filter paper, rinsed three times with sterile distilled water to remove residual calcium ions, and subsequently dried using hot air drying at 50–70 °C for 4–5 h. The encapsulation spore preparations were stored at 4 °C for further experiments.

### 2.3. Rearing Conditions

Three replicate tanks were assigned to each dietary treatment, with 20 fish stocked per tank. Experimental diets were administered twice daily at 09:00 and 16:00 during a eight-week feeding trial (from October to December 2025). Fish were observed for 30 min after each feeding to ensure the absence of uneaten feed. Any remaining feed was collected and weighed at the end of each day to estimate feed requirements for the subsequent day. The average feed intake for juvenile Nile tilapia was approximately 4% of body weight throughout the experimental period.

Water quality parameters were monitored and maintained within optimal ranges for Nile tilapia throughout the experiment [43]. These parameters included temperature (28.5 °C), pH (7.8), dissolved oxygen (5.76 mg/L), and NH_3_ (0.15 mg/L).

### 2.4. Sample Collection

Fish were weighed at two-week intervals to assess growth performance. Mortality was recorded daily, and dead fish were promptly removed from the tanks. Survival rate was calculated at weeks 2 and 4 to determine cumulative survival.

At the end of the 8-week feeding trial, three fish per replicate tank were randomly selected and anesthetized with clove oil [44]. Briefly, the stock solution was diluted in tank water to achieve a working concentration of 100 mg/L. Individual fish were immersed in the anesthetic solution until complete sedation was conformed. Blood and tissue samples were then collected for subsequent analyses. Blood was drawn from the caudal vein using a 1 mL syringe, allowed to clot at 4 °C for 4 h, and centrifuged at 1000× *g* for 10 min at 4 °C to obtain serum for antioxidant analysis [45].

Mid-intestinal tissue samples (approximately 50 mg) were collected, placed into sterile tubes containing 200 µL of TRIzol™ reagent (Invitrogen™, Life Technologies, Carlsbad, CA, USA), and stored at −80 °C for gene expression analysis. In addition, midgut and liver tissues were fixed in 10% neutral buffered formalin for histological examination. The distal intestine was collected for total bacterial count analysis and stored in sterile 0.85% (*w*/*v*) NaCl solution until processing.

### 2.5. Growth Performance Indices

Fish growth performance was assessed using the following indices according to previously described methods [45,46]:

- Weight gain (WG, %) = [(Final body weight − Initial weight)/Initial weight] × 100.
- Specific growth rate (SGR, %) = [ln (Final weight) − ln (Initial weight)]/days × 100.
- Feed conversion ratio (FCR) = [Feed consumed (g)/Weight gain (g)].
- Daily weight gain (DWG, g/day) = (Final body weight − Initial body weight)/days.
- Periodic growth rate (PWG, g/day) = [(Final weight of period − Initial weight of the period)/period day].

### 2.6. Blood Biochemical Analysis

#### 2.6.1. ABTS Radical Scavenging Activity

Antioxidant activity was determined using the ABTS radical scavenging assay following the method described by Boonkong, et al. [47] with minor modification. Briefly, 20 µL of serum sample was mixed with 200 µL of 2,2′-Azino-bis(3-ethylbenzothiazoline-6-sulphonic acid) (ABTS) solution. The mixture was gently vortexed and incubated in the dark at room temperature for 10 min. Absorbance was measured at 734 nm using a microplate reader. Antioxidant activity was quantified by comparison with a Trolox standard curve ranging from 0 to 2.5 mg/mL.

#### 2.6.2. Superoxide Dismutase Activity

Superoxide dismutase (SOD) activity was measured using a commercial assay kit (CheKine™ Micro Superoxide Dismutase Activity Assay Kit, KTB1030; Sigma-Aldrich, St. Louis, MO, USA) according to the manufacturer’s instructions. Serum samples were incubated with the provided reagents, and absorbance was recorded at 450 nm using a Varioskan LUX microplate reader (Thermo Scientific, Vantaa, Finland) [48].

#### 2.6.3. Malondialdehyde Assay

Lipid peroxidation was assessed by measuring malondialdehyde (MDA) levels following the method of Karatas and EM [49]. Briefly, 50 µL of serum was mixed with 100 µL of thiobarbituric acid (TBA) reagent consisting of 0.375% TBA and 0.25 N HCl in 15% trichloroacetic acid (TCA). The mixture was incubated at room temperature for 5 min and then heated at 95 °C for 5 min. After cooling on ice, absorbance was measured at 532 nm. MDA concentration was calculated using a standard curve ranging from 0 to 300 µmol/L.

### 2.7. Histological Analysis

For each treatment group, anterior intestinal and liver tissues (*n* = 5) were collected and subjected to histological processing. The samples were first fixed and then dehydrated through a sequential ethanol gradient consisting of 10%, 20%, 30%, 50%, 70%, and 100% ethanol. Following dehydration, the tissues were clarified by two immersions in xylene, each lasting 60 min.

Subsequently, the specimens were infiltrated with a transitional mixture of xylene and paraffin (1:2, *v*/*v*) prior to paraffin processing. This was followed by two successive paraffin infiltration steps, after which the tissues were maintained in molten paraffin overnight before being embedded into paraffin blocks.

Paraffin-embedded samples were sectioned at a thickness of 5 μm using a rotary microtome (Leica 2025, Wetzlar, Germany). The sections were stained with hematoxylin and eosin (H&E; Solarbio, G1120, Beijing, China) to evaluate tissue architecture and cellular characteristics. Microscopic examination was carried out using a CX43 light microscope (Olympus, Hachioji-shi, Tokyo, Japan) fitted with an E620 digital imaging system. Morphometric measurements were performed according to the methodological framework described by previous studies [45,50].

### 2.8. Intestinal Bacterial and Yeast Counts

The intestine was thoroughly washed with sterile saline solution (0.85% NaCl) and homogenized for the determination of total bacterial count, total lactic acid bacteria (LAB), and total yeast count using the tenfold serial dilution method. Approximately 1 g of intestinal tissue was homogenized in 9 mL of sterile saline to obtain an initial 10^−1^ dilution. Tenfold serial dilutions were subsequently prepared up to 10^−6^. For each appropriate dilution, 0.1 mL aliquots were spread-plated in triplicate onto selective media. Plate count agar (PCA; HiMedia Laboratories, Mumbai, India) was used for enumeration of total bacteria, De Man, Rogosa and Sharpe (MRS) agar (HiMedia Laboratories, India) for total LAB, and potato dextrose agar (PDA; HiMedia Laboratories, India) for total yeast. PCA and MRS plates were incubated at 37 °C for 24–48 h under aerobic conditions, whereas PDA plates were incubated at 28 °C for 72 h. Plates containing 30–300 colonies were selected for counting. Colony-forming units were calculated by multiplying the colony number by the corresponding dilution factor and expressed as log_10_ CFU/g of intestinal tissue.

### 2.9. Gene Expression by Quantitative PCR (qPCR)

Total RNA was extracted from mid-intestinal tissue samples using TRIzol™ reagent following homogenization with a Bullet Blender^®^ homogenizer (Next Advance, New York, NY, USA) based on previous studies [51]. Homogenized samples were incubated at room temperature for 2–3 min, after which 100 µL of chloroform was added. The mixtures were vortexed briefly, incubated for 2 min, and centrifuged at 12,000 rpm for 15 min at 4 °C. The aqueous phase containing RNA was collected and further purified using a Total RNA extraction kit (Omega Bio-tek, Norcross, GA, USA) according to the manufacturer’s instructions. RNA concentration and purity were assessed using a NanoDrop™ One/OneC spectrophotometer (Thermo Fisher Scientific, Waltham, MA, USA) [52].

Complementary DNA (cDNA) was synthesized from 1 µg of total RNA using a reverse transcription kit (BIO-RAD, Hercules, CA, USA). Quantitative real-time PCR (qPCR) was conducted in a 20 µL reaction volume containing 100 ng of cDNA, 0.4 µL of each primer (10 µM), and 10 µL of 2× iTaq Universal SYBR Green Supermix (BIO-RAD, USA). Amplification was performed using a CFX Connect™ Real-Time PCR Detection System (BIO-RAD, USA) under the following conditions: initial denaturation at 95 °C for 30 s, followed by 40 cycles of 95 °C for 15 s and 60 °C for 30 s [45]. A melt curve analysis was included to confirm amplification specificity. Relative gene expression levels were calculated using the 2^−ΔΔCt^ method [53], with *β-actin* used as the reference gene. All the primers were adopted from Sheng, et al. [54] study. Primer sequences are provided in Table 1.

### 2.10. Aeromonas hydrophila Challenge Test

*Aeromonas hydrophila* used in the present study was cultured in tryptic soy broth (HiMedia, India) at 28 °C for 16 h. Bacterial cells were harvested by centrifugation at 12,000 × *g* for 15 min at 4 °C, washed three times with sterile phosphate-buffered saline (PBS), and resuspended in sterile PBS. Bacterial concentration was determined by tenfold serial dilution and plate counting on tryptic soy agar plates.

Fish in the negative control group were injected intraperitoneally with sterile PBS, whereas fish in the remaining treatments were injected with *A. hydrophila*. All challenged fish were intraperitoneally injected with *A. hydrophila* at a dose of 2.5 × 10^6^ CFU/g body weight, following a previously described protocol [55]. After challenge, fish were monitored daily, and cumulative mortality was recorded over a 14-day period.

After the challenge trial, surviving and moribund Nile tilapia were managed under strict biosecurity and hygienic disposal procedures to prevent environmental release of the pathogen. All remaining fish were humanely euthanized using an overdose of tricaine methanesulfonate (MS-222; 300 mg/L) for the euthanasia of experimental fish [56]. Death was confirmed by cessation of opercular movement and absence of reflex responses before further handling. All dead and euthanized fish were collected in sealed, leakproof biosecure containers and transported to a licensed facility for high-temperature incineration, consistent with aquatic animal health and biosafety recommendations [57]. In addition, tanks, equipment, and effluent water were disinfected with sodium hypochlorite before disposal, in accordance with established aquaculture biosecurity protocols.

### 2.11. Statistical Analysis

Prior to statistical analysis, microbial count data were log-transformed to satisfy assumptions of normality and homoscedasticity. Data normality was assessed using the Shapiro–Wilk test [58]. Differences among treatments were analyzed by one-way analysis of variance (ANOVA), followed by Duncan’s multiple range test using SPSS software (version 29.0.2.0; IBM Corp., Armonk, NY, USA). In addition, fish survival during the 14-day post-challenge period following *A. hydrophila* injection was analyzed using Kaplan–Meier survival curves, and differences among treatments were evaluated with the log-rank test. Pearson correlation analysis and principal component analysis (PCA) were conducted using standardized (z-score transformed) datasets including all quantitative indicators measured in this study (growth performance indices, antioxidant parameters, intestinal microbial counts, histological parameters and immune-related gene expression levels). Variables included in PCA were screened to ensure complete datasets (no missing values) and adequate variance (variance > 1 after scaling). PCA suitability was verified using the Kaiser–Meyer–Olkin (KMO) measure of sampling adequacy and Bartlett’s test of sphericity prior to dimensionality reduction. Principal components with eigenvalues > 1 were retained for interpretation. All statistical analyses and graphical visualizations were conducted using R software (version 4.3.1; R Foundation for Statistical Computing, Vienna, Austria). The following R packages were used as *stats* for correlation analysis, *FactoMineR* and *factoextra* for PCA, *survival* and *survminer* for survival analysis, and *ggplot2* for data visualization. Results are expressed as mean ± standard deviation. Statistical significance was accepted at *p* < 0.05.

## 3. Results

### 3.1. Growth Performance Analysis

*Lysinibacillus* sp. PWR01 encapsulation concentration affected growth performance from week 4 onward, while survival remained unchanged throughout the experiment (Table 2). At week 4, FW ranged from 26.00 g in the control to 27.63 g at 10^5^ CFU/g. WG (15.18–16.87 g), PWG (137.83–156.66%), SGR (2.92–3.14%/day), and DWG (0.54–0.60 g/day) showed higher mean values at 10^5^–10^6^ CFU/g than in the control. FCR at week 4 was similar among treatments (0.83–0.84). However, these parameters have no different significant among treatments.

**Figure 2:**
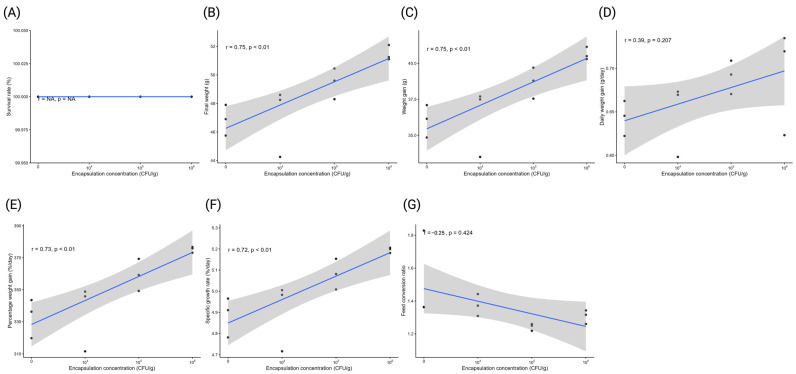
Correlations between encapsulation concentration (CFU/g) and growth-related parameters measured at week 8: (**A**) survival rate, (**B**) final weight, (**C**) weight gain, (**D**) daily weight gain, (**E**) percent weight gain, (**F**) specific growth rate, and (**G**) feed conversion ratio. Solid lines indicate good linear regression fit and shaded areas represent 95% confidence intervals. Pearson correlation coefficients (r) and *p*-values are shown in each panel.

At week 8, FW increased significantly with encapsulation concentration (r = 0.75, *p* < 0.01) (Figure 2), reaching 51.48 g at 10^6^ CFU/g compared with 46.85 g in the control, representing a 9.88% increase relative to the control. WG was also positively correlated with concentration (r = 0.75, *p* < 0.01), increasing from 36.03 g in the control to 40.65 g at 10^6^ CFU/g, corresponding to a 12.82% improvement. PWG showed a strong positive correlation (r = 0.73, *p* < 0.01), ranging from 333.17% to 375.23% across the same treatments (+12.62% compared with control). SGR increased with encapsulation concentration (r = 0.72, *p* < 0.01), with the highest value observed at 10^6^ CFU/g (5.20%/day), 6.34% higher than the control (4.89%/day). DWG exhibited a positive but non-significant association with concentration (r = 0.39, *p* = 0.21), reaching 0.73 g/day at 10^6^ CFU/g, which was 14.06% greater than the control (0.64 g/day). FCR showed a negative, non-significant correlation with encapsulation concentration (r = −0.25, *p* = 0.42), with lower values at 10^5^–10^6^ CFU/g (1.24–1.31) than in the control (1.52), indicating an improvement in feed efficiency of up to 18.42% at 10^5^ CFU/g.

### 3.2. Antioxidant and Lipid Peroxidation Analysis

**Figure 3:**
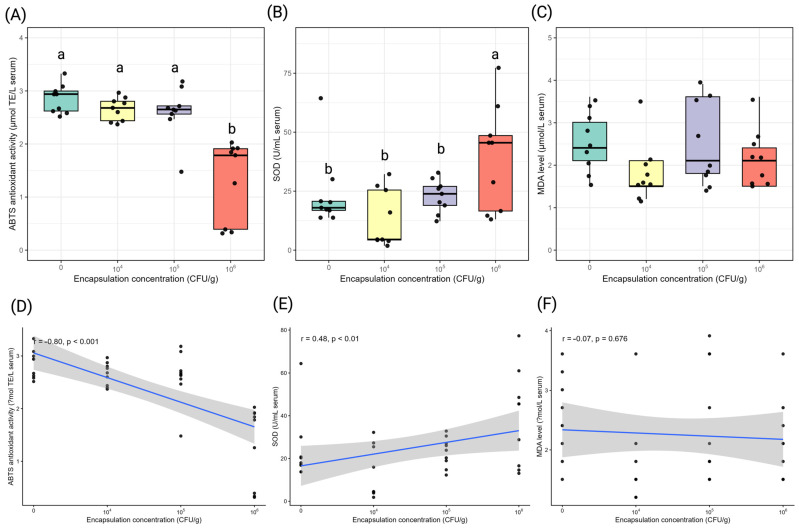
Effects of *Lysinibacillus* sp. PWR01 encapsulation concentration (CFU/g) on blood serum oxidative stress and antioxidant parameters of Nile tilapia (*Oreochromis niloticus*). (**A**) ABTS radical scavenging activity (μmol TE/L), (**B**) SOD activity (U/mL), and (**C**) MDA level (μmol/L). Different lowercase letters indicate significant differences among treatments (*p* < 0.05). Pearson correlation analyses between encapsulation concentration and (**D**) ABTS activity, (**E**) SOD activity, and (**F**) MDA level.

Encapsulation concentration significantly altered antioxidant enzyme activity and radical scavenging capacity, while lipid peroxidation remained unchanged (Figure 3).

Relative to the control, serum ABTS activity declined at the highest encapsulation level. ABTS activity was comparable among the control, 10^4^ CFU/g, and 10^5^ CFU/g groups (2.61–2.85 μmol TE/L), but was significantly lower at 10^6^ CFU/g (1.31 μmol TE/L; *p* < 0.05). Specifically, ABTS activity at 10^6^ CFU/g was approximately 2.0-fold lower than the control. A strong negative correlation was observed between encapsulation concentration and ABTS activity (r = −0.80, *p* < 0.001).

Serum SOD activity increased with encapsulation concentration. Mean SOD activity ranged from 13.34 U/mL at 10^4^ CFU/g to 38.99 U/mL at 10^6^ CFU/g. At 10^6^ CFU/g, SOD activity was approximately 2.9-fold higher than the lowest-dose group (10^4^ CFU/g) and markedly elevated relative to the control (*p* < 0.05). SOD activity showed a significant positive correlation with encapsulation concentration (r = 0.48, *p* < 0.01).

MDA levels did not differ significantly among treatments, ranging from 1.84 to 2.54 μmol/L. No significant correlation was detected between encapsulation concentration and MDA level (r = −0.07, *p* = 0.68).

### 3.3. Microbial Counts Analysis

**Figure 4:**
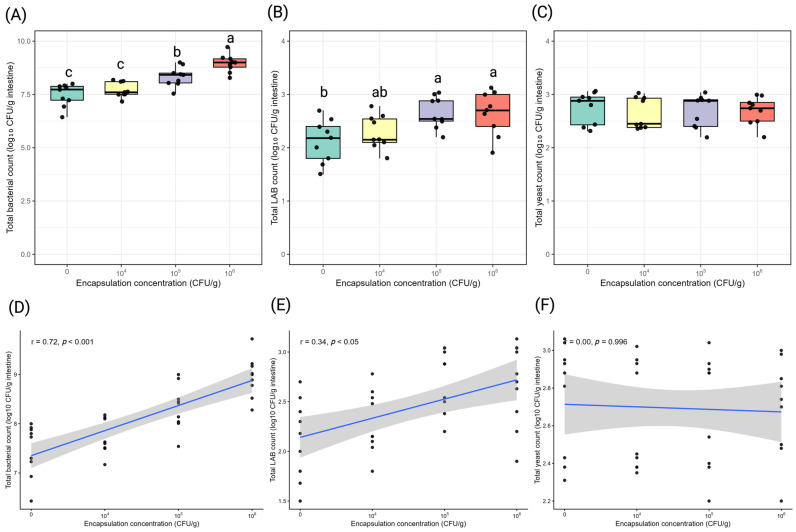
Effects of *Lysinibacillus* sp. PWR01 encapsulation concentration (CFU/g) on Nile tilapia (*Oreochromis niloticus*) intestinal microbial populations. (**A**) Total bacterial count, (**B**) total LAB count, and (**C**) total yeast count. Different lowercase letters indicate significant differences among treatments (*p* < 0.05). Pearson correlation analyses between encapsulation concentration and (**D**) total bacterial count, (**E**) LAB count, and (**F**) yeast count. Dots represent individual data points.

Encapsulation concentration significantly influenced total intestinal bacterial and LAB counts, whereas yeast counts were unaffected (Figure 4).

Total intestinal bacterial count increased progressively with encapsulation concentration. Mean bacterial counts rose from 7.47 log_10_ CFU/g intestine in the control group to 7.70 log_10_ CFU/g at 10^4^ CFU/g, 8.34 log_10_ CFU/g at 10^5^ CFU/g, and 8.96 log_10_ CFU/g at 10^6^ CFU/g (*p* < 0.05). Compared with the control, total bacterial abundance increased by 0.87, and 1.49 log units at 10^5^, and 10^6^ CFU/g, respectively. Encapsulation concentration showed a strong positive correlation with total bacterial count (r = 0.72, *p* < 0.001).

Total LAB count also differed significantly among treatments. LAB counts increased from 2.12 log_10_ CFU/g intestine in the control to 2.29 log_10_ CFU/g at 10^4^ CFU/g and peaked at 2.66 log_10_ CFU/g at 10^5^ CFU/g, with a similar value at 10^6^ CFU/g (2.64 log_10_ CFU/g; *p* < 0.05). Relative to the control, LAB abundance increased by 0.54, and 0.52 log units at 10^5^, and 10^6^ CFU/g, respectively. A positive correlation was observed between encapsulation concentration and LAB count (r = 0.34, *p* < 0.05).

Total intestinal yeast count remained stable across treatments, ranging from 2.64 to 2.75 log_10_ CFU/g intestine, with no significant differences among encapsulation concentrations. In addition, no significant correlation was detected between encapsulation concentration and yeast count.

### 3.4. Histological Characteristics

**Figure 5:**
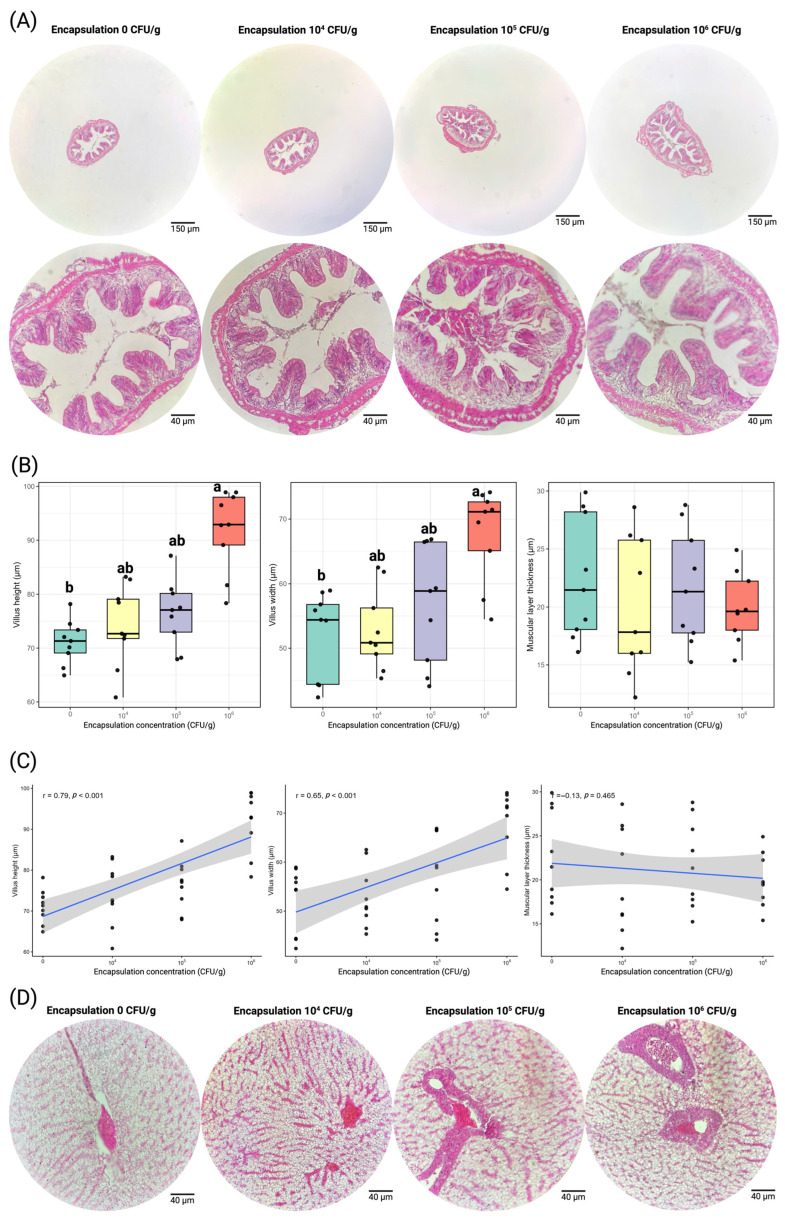
Histological structure and morphometric characteristics of Nile tilapia (*Oreochromis niloticus*) intestinal and hepatic tissues under different *Lysinibacillus* sp. PWR01 encapsulation concentrations (CFU/g). (**A**) Representative H&E-stained cross-sections of the anterior intestine at encapsulation levels of 0, 10^4^, 10^5^, and 10^6^ CFU/g. (**B**) Boxplots showing villus height, villus width, and muscular layer thickness across treatments; different letters indicate significant differences (*p* < 0.05). (**C**) Correlation between encapsulation concentration and intestinal morphometric parameters. (**D**) Representative H&E-stained liver sections illustrating hepatocyte organization, sinusoidal spaces, and central vein structure across treatments. Scale bars: 150 µm (upper intestinal overview), 40 µm (intestinal and liver details). Dots represent individual data points.

Encapsulation supplementation significantly increased villus height and villus width, while muscular layer thickness remained unchanged and liver histology exhibited normal structural organization across all treatments (Figure 5).

Mean villus height increased progressively with encapsulation concentration, ranging from 71.10 µm in the control group to 91.90 µm in the 10^6^ CFU/g treatment (Figure 5B). The highest supplementation level produced significantly greater villus height than the control (*p* < 0.05), whereas the 10^4^ and 10^5^ CFU/g treatments showed intermediate values without significant differences from adjacent groups (Figure 5B). Correlation analysis indicated a strong positive association between encapsulation concentration and villus height (r = 0.79, *p* < 0.001) (Figure 5C).

Villus width showed a similar trend. Mean values increased from 52.22 µm in the control group to 67.74 µm at 10^6^ CFU/g (Figure 5B). The 10^6^ CFU/g treatment exhibited significantly greater villus width compared with the control (*p* < 0.05), while the 10^4^ and 10^5^ CFU/g treatments displayed intermediate measurements without significant differences (Figure 5B). A significant positive correlation was detected between encapsulation concentration and villus width (r = 0.65, *p* < 0.001) (Figure 5C).

Muscular layer thickness varied within a narrow range among treatments, from 19.96 µm to 22.44 µm (Figure 5B). No statistically significant differences were detected among groups (*p* > 0.05). The correlation between encapsulation concentration and muscular thickness was not significant (r = −0.13, *p* = 0.465) (Figure 5C).

Histological examination of the liver revealed well-preserved hepatic architecture in all treatments (Figure 5D). Hepatocytes were arranged in typical cords radiating from the central vein and separated by clearly visible sinusoidal spaces. Hepatocyte cytoplasm appeared homogeneous with centrally located nuclei, and no cellular degeneration, vacuolation, or structural disruption was observed across treatments. Sinusoidal structures remained regular and continuous, and the central vein maintained normal morphology without dilation or congestion. No necrotic areas, inflammatory cell infiltration, or tissue disorganization were detected in any group (Figure 5D). The structural integrity of hepatocytes and sinusoidal networks was comparable among all encapsulation concentrations.

### 3.5. Gene Expression

**Figure 6:**
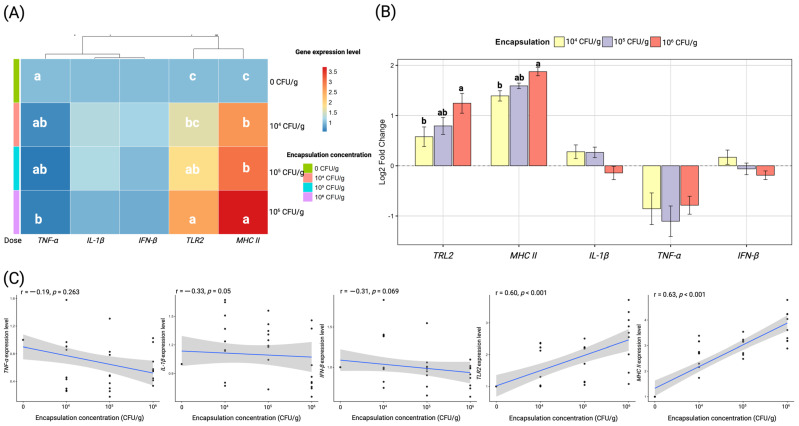
Dose-dependent effects of encapsulated *Lysinibacillus* sp. PWR01 on immune-related gene expression. (**A**) Heatmap of relative transcript levels (*TNF-α*, *IL-1β*, *IFN-β*, *TLR2*, and *MHC II)* across treatments (0, 10^4^, 10^5^, and 10^6^ CFU/g). (**B**) Log_2_ fold changes (mean ± SE) relative to the control. (**C**) Pearson correlations between encapsulation concentration and gene expression levels. Different lowercase letters indicate significant differences among treatments (*p* < 0.05). Dots represent individual data points.

Encapsulation increased the transcript levels of *TLR2* and *MHC II*, while *IL-1β*, *TNF-α*, and *IFN-β* showed no consistent upregulation across doses (Figure 6). At 10^4^–10^6^ CFU/g, *TLR2* transcripts significantly increased from the control (1.00) to 1.61–2.53, respectively, corresponding to log_2_ fold changes of 0.58–1.25. *MHC II* transcripts significantly rose from 1.00 in the control to 2.67–3.71 at 10^4^–10^6^ CFU/g, with log_2_ fold changes of 1.39–1.87, respectively. In contrast, *IL-1β* transcript levels were 1.26–0.94 at 10^4^–10^6^ CFU/g, yielding log_2_ fold changes of 0.28–−0.14. *TNF-α* transcripts decreased relative to the control to 0.67, 0.56 and 0.62 at 10^4^–10^6^ CFU/g, corresponding to log_2_ fold changes of −0.86, −1.10, and −0.79. *IFN-β* transcript levels were 1.17, 0.98, and 0.89 at 10^4^–10^6^ CFU/g, with log_2_ fold changes of 0.17, −0.06, and −0.19.

Pearson correlation analysis showed positive associations between encapsulation concentration and *TLR2* (r = 0.60, *p* < 0.001) and *MHC II* expression (r = 0.63, *p* < 0.001), whereas correlations with *IL-1β*, *TNF-α*, and *IFN-β* were not significant (*p* ≥ 0.05).

### 3.6. Pearson Correlation and PCA

**Figure 7:**
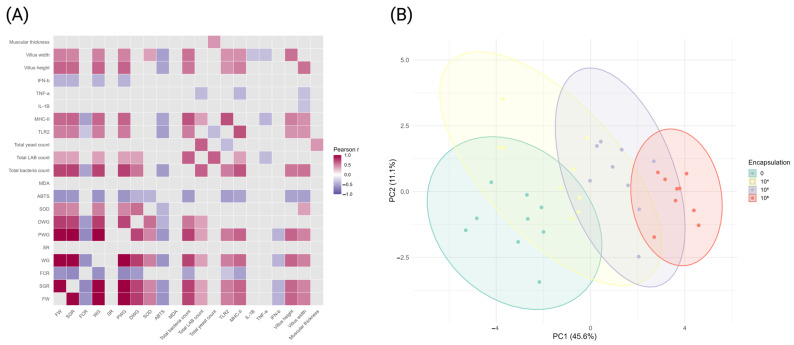
Multivariate relationships among growth performance, antioxidant status, microbiota composition, and immune gene expression in response to encapsulation. (**A**) Pearson correlation heatmap showing significant pairwise correlations (*p* < 0.05) among growth indices, antioxidant parameters, intestinal microbiota, and immune-related genes; correlation coefficients (r) are color-coded from negative (blue) to positive (red). (**B**) Principal component analysis score plot based on all measured variables, showing sample distribution according to encapsulation levels (0, 10^4^, 10^5^, and 10^6^ CFU/g); ellipses represent 95% confidence intervals for each group.

Multivariate analysis showed significant associations among growth, antioxidant, microbiota, and immune variables and a clear separation of samples by encapsulation level (Figure 7). The Pearson correlation heatmap identified significant positive and negative correlations (*p* < 0.05) among multiple parameters, while self-correlations were excluded and non-significant relationships were masked. Positive correlations (r > 0.50, *p* < 0.05) were observed between growth-related indices (FW, WG, DWG, and PWG) and immune markers TLR2 and MHC II, whereas negative correlations (r < −0.50, *p* < 0.05) were detected between FCR and these growth indices. Microbiota variables (total bacteria, total LAB, and total yeast) showed significant positive correlations with TLR2 and MHC II (r = 0.48–0.67, *p* < 0.05), while TNF-α was negatively correlated with several growth and microbiota parameters (r = −0.41 to −0.60, *p* < 0.05). Principal component analysis explained 56.7% of the total variance on the first two axes, with PC1 accounting for 45.6% and PC2 for 11.1%. Samples were separated along PC1 according to encapsulation levels, with minimal overlap between 0 CFU/g and 10^6^ CFU/g groups, whereas intermediate overlap was observed between 10^4^ and 10^5^ CFU/g groups.

### 3.7. Post-Challenge Survival Following A. hydrophila Infection

**Figure 8:**
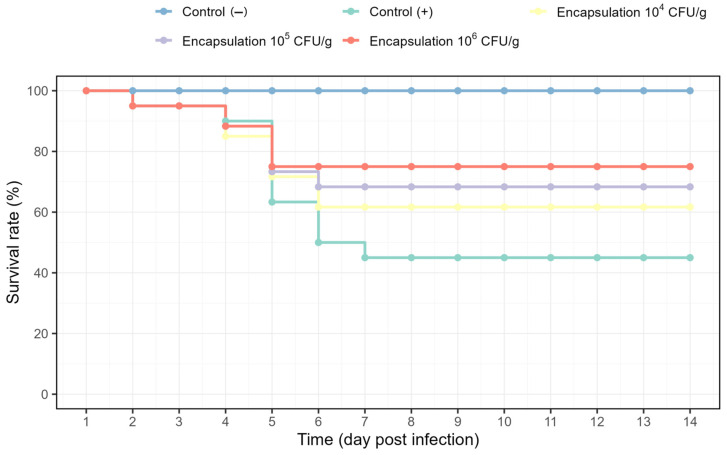
Survival rate (%) of Nile tilapia (*Oreochromis niloticus*) over 14 days following *Aeromonas hydrophila* challenge under different treatments.

Encapsulation concentration significantly affected survival following *A. hydrophila* challenge (Figure 8). Survival in the negative control remained at 100% throughout the 14-day post-challenge period. In contrast, survival in the positive control declined rapidly after day 4, reaching 63.33% on day 5 and stabilizing at 45.00% from day 7 to day 14. Fish receiving encapsulated *Lysinibacillus* treatments showed a delayed and attenuated mortality pattern compared with the positive control. In the 10^6^ CFU/g group, mortality increased modestly around days 4–5 but stabilized earlier (by day 6), reaching a final survival rate of 75.00%. Notably, the mortality peak in the 10^6^ CFU/g group occurred within the same early window (days 4–5) but ended more rapidly and at a lower magnitude than in the positive control, suggesting improved early-phase resistance to bacterial proliferation. Similarly, final survival rates were 68.33% and 61.67% for the 10^5^ and 10^4^ CFU/g groups, respectively, all exceeding that of the positive control (45.00%). The progressive improvement in survival from 10^4^ to 10^6^ CFU/g demonstrates a dose-dependent protective effect, although the survival curves converged after day 6, indicating that the principal benefit was exerted during the acute infection phase.

In addition, Chi-square analysis showed a significant difference in survival between the positive and negative controls (χ^2^ = 50.132, *p* < 0.001; Table 3). Survival in the 10^5^ CFU/g (χ^2^ = 4.226, *p* = 0.040) and 10^6^ CFU/g (χ^2^ = 5.719, *p* = 0.017) groups differed significantly from the positive control, whereas the 10^4^ CFU/g group did not (χ^2^ = 1.454, *p* = 0.228; Table 3). No significant differences were detected among the encapsulation treatments (χ^2^ ≤ 1.398, *p* ≥ 0.237).

## 4. Discussion

### 4.1. Growth Performance and Feed Utilization

The present findings demonstrate that encapsulated *Lysinibacillus* sp. PWR01 can significantly enhance fish growth performance, particularly over an extended feeding duration. By week 8, fish fed diets with higher probiotic encapsulation (10^5^–10^6^ CFU/g) showed greater FW, WG, PWG, and SGR compared to the control, with a clear dose-dependent trend (correlations r ≈ 0.75, *p* < 0.01). Notably, improvements were modest and statistically non-significant at week 4, suggesting that the probiotic’s benefits manifest more strongly after sustained administration. This time-lag may reflect the period required for the probiotic to colonize the gut or modulate host physiology. Similar delayed growth enhancements have been observed in other studies. For example, feeding *L. sphaericus* to zebrafish over 33 days led to increasing WG and SGR with rising dose [30]. Notably, even at the highest tested concentration (10^7^ CFU/mL), growth parameters did not differ statistically from controls. By contrast, the present study achieved statistically significant growth enhancement at 10^6^ CFU/g, indicating that effective dose thresholds may be reduced through encapsulation, likely due to improved gastrointestinal stability and delivery efficiency. Similarly, Mani, et al. [33] reported that dietary supplementation with *L. macroides* at 10^7^–10^8^ CFU/mL significantly improved growth and feed conversion ratio in *Cyprinus carpio* fingerlings. However, the probiotic was administered in pelletized form without protective encapsulation, and relatively higher microbial loads were required to achieve statistical significance. When contrasted with these findings, the current encapsulated 10^6^ CFU/g treatment achieved comparable improvements in FW and SGR at a lower concentration range. In Nile tilapia, diets supplemented related spore-formers (*Bacillus* spp.) over 8–10 weeks have consistently improved WG and feed conversion efficiency. For instance, *Bacillus subtilis* at 10^10^ CFU/g halved the FCR and boosted WG by approximately 40% relative to un-supplemented fish [59]. In the current study, FCR likewise tended to decrease, although this drop was not statistically significant. The overall improvement in growth metrics without adverse effects on survival or body condition aligns with a broad literature reporting that probiotics enhance nutrient utilization and growth in diverse aquaculture species [14,60]. The mechanisms likely involve probiotics contributing exogenous digestive enzymes and vitamins, as well as stimulating the host’s digestive capacity [61]. Indeed, encapsulated probiotics have been shown to increase intestinal villi height and digestive enzyme activity, thereby increasing nutrient absorption in species like tilapia [62,63]. Enhanced feed intake or appetite regulation may also play a role; one study on encapsulated *Lactobacillus* noted upregulation of appetite-related genes (e.g., ghrelin) in fish fed the probiotic [64]. Collectively, the growth promotion seen with *Lysinibacillus* sp. PWR01 treatment is consistent with these reports. The lack of significant differences at early time points and the convergence of growth curves by week 8 in the high-dose groups further suggest that while low doses may be insufficient for measurable impact, an adequate concentration sustained over several weeks is required to realize probiotic growth benefits in aquaculture.

### 4.2. Antioxidant Status and Lipid Peroxidation of Blood Serum

Dietary *Lysinibacillus* encapsulation elicited notable changes in the antioxidant defense parameters of the fish. Serum SOD activity was markedly elevated in probiotic-fed groups, reaching nearly 3-fold higher levels at 10^6^ CFU/g compared to controls. This positive correlation between dose and SOD activity (r = 0.48, *p* < 0.01) indicates an enhanced enzymatic capacity to neutralize superoxide radicals. Concomitantly, MDA concentration remained unchanged across treatments, suggesting that higher probiotic levels prevented any rise in oxidative stress (and may have helped maintain MDA at baseline levels). These outcomes align with recent probiotic studies reporting bolstered antioxidant defenses in fish. For instance, zebrafish receiving *L. sphaericus* exhibited significantly increased SOD and catalase activities coupled with a linear decrease in MDA content as the probiotic dose rose [30]. Likewise, tilapia fed high-dose *B. subtilis* showed elevated SOD, catalase, and glutathione peroxidase activities and significantly lower MDA than controls [59]. The general interpretation is that probiotics can induce the host’s antioxidant enzyme systems, thereby limiting lipid peroxidation and cellular damage under physiological or stress conditions [65,66]. Interestingly, in the present study the total radical scavenging capacity (ABTS assay) of serum was lower in the high-dose group. This inverse relationship with probiotic dose (r ≈ −0.80) is somewhat counterintuitive, as one might expect overall antioxidant capacity to increase. A possible explanation is that the heightened SOD activity rapidly neutralized free radicals [67,68,69], thus leaving fewer circulating antioxidant compounds detectable by the ABTS assay. Regardless, the fish did not show elevated MDA, implying no net increase in oxidative damage despite the ABTS reduction. Previous work has noted that probiotic-fed fish often experience less oxidative stress during challenges or high metabolic demand. For example, probiotic-driven activation of the Nrf2 antioxidant pathway has been suggested as a mechanism in carp and trout, leading to strengthened endogenous antioxidant responses [70]. The present results support the view that *Lysinibacillus* supplementation can fortify antioxidant defenses (notably SOD), potentially conferring greater cellular protection. This antioxidant boost is beneficial for overall health and may contribute to the improved growth and disease resistance observed, since oxidative stress is known to impair growth and immune function in fish.

### 4.3. Intestinal Microbial Counts

Encapsulation of *Lysinibacillus* sp. PWR01 had a pronounced impact on the gut microbiota, as reflected by increased counts of total cultivable bacteria and LAB in the intestine. Total bacterial counts rose steadily with probiotic dose, which was nearly an order of magnitude higher at 10^6^ CFU/g than in the control. In addition, LAB counts also increased significantly. These shifts suggest that the probiotic either directly contributed to these populations (as *Lysinibacillus* itself is a Gram-positive fermentative bacterium) or created gut conditions favorable to indigenous beneficial microbes. No changes were seen in intestinal yeast counts, indicating the effect was specific to the bacterial microbiota. The enrichment of LAB is particularly noteworthy, as lactic acid bacteria are often associated with improved gut health and pathogen resistance in fish [71]. A number of studies have likewise reported that probiotic supplements can modulate the gut microbial community toward a more favorable profile. Qiu, et al. [30] found that zebrafish fed *L. sphaericus* exhibited higher abundances of beneficial genera (e.g., *Cetobacterium*, *Lactobacillus*) and a significant reduction in the relative abundance of the pathogenic genus *Aeromonas*. In our challenge experiment, fish receiving *Lysinibacillus* had improved survival to *Aeromonas* infection, and it is tempting to link this to potential suppression of *Aeromonas* in the gut microbiome as seen in Qiu, et al. [30] work. The likely mechanisms for these microbiota changes include competitive exclusion and antimicrobial production. Probiotic *Lysinibacillus* are known to inhibit pathogens by secreting antagonistic compounds and by outcompeting harmful bacteria for nutrients and adhesion sites on the intestinal mucosa. A recent isolation study in marine fish showed a *Lysinibacillus* strain could strongly inhibit *Vibrio* pathogens and co-aggregate with pathogenic bacteria, thereby preventing their colonization [31]. In the present context, *Lysinibacillus* sp. PWR01 may have directly suppressed opportunistic gut pathogens, allowing total bacterial counts to rise and beneficial commensals (like LAB) to flourish.

### 4.4. Histological Characteristics

Histological observations demonstrated that dietary encapsulated *Lysinibacillus* influenced intestinal morphology while maintaining normal hepatic structure. It can be seen that in the anterior intestine, fish receiving higher encapsulation levels exhibited increased villus height and width, particularly in the 10^6^ CFU/g treatment. These structural modifications indicate an expansion of the absorptive surface area, which is widely recognized as a key determinant of nutrient uptake efficiency in fish [72,73,74]. Enlarged villi increase the contact interface between digested nutrients and the intestinal epithelium, thereby facilitating more efficient absorption and assimilation [72,75]. Similar morphological responses have been reported in several probiotic studies in aquaculture. For example, dietary supplementation with *Bacillus subtilis* in Nile tilapia significantly increased villus height and intestinal fold development, resulting in improved digestive efficiency and growth performance [59,76]. Likewise, probiotics based on *Lactobacillus* or *Bacillus* species have been shown to enhance intestinal villus architecture in carp and tilapia, which was associated with improved feed utilization and growth outcomes [59,76,77,78]. The positive correlations observed between encapsulation concentration and villus dimensions in the present study therefore align with the improved growth performance recorded during the feeding trial. Interestingly, muscular layer thickness did not differ significantly among treatments. This indicates that the probiotic primarily affected mucosal and absorptive structures rather than deeper muscular tissues. The mechanisms underlying these morphological improvements may involve several probiotic-mediated processes. Beneficial bacteria can stimulate epithelial cell proliferation, enhance mucosal integrity, and modulate gut-associated immune responses [79,80,81]. In particular, spore-forming bacteria such as *Lysinibacillus* and *Bacillus* are known to produce extracellular enzymes and bioactive metabolites that promote digestive activity and intestinal health [32,82]. Additionally, probiotics may regulate gut microbiota composition, leading to increased populations of beneficial bacteria such as lactic acid bacteria, which in turn produce short-chain fatty acids and other metabolites that support intestinal epithelial development [83,84,85]. The increase in LAB counts observed in the present study is therefore consistent with these morphological findings and suggests that microbial modulation may have contributed to the enhanced intestinal structure.

In addition to intestinal effects, liver histology revealed well-preserved hepatic architecture across all treatments. Hepatocytes were arranged in typical radial cords surrounding the central vein, and sinusoidal spaces were clearly defined. No signs of hepatocellular degeneration, necrosis, inflammatory infiltration, or lipid accumulation were observed. These findings indicate that dietary encapsulated *Lysinibacillus* did not exert hepatotoxic effects even at the highest supplementation level. The preservation of liver integrity may also be linked to the enhanced antioxidant status observed in probiotic-fed fish. Increased SOD activity suggests improved protection against oxidative stress [86], which is known to contribute to hepatocellular damage in fish exposed to environmental or metabolic stressors. By strengthening antioxidant defenses, probiotics may help maintain cellular stability and prevent oxidative injury to hepatic tissues [86,87]. The stable MDA levels observed in the present study further support the absence of oxidative damage in hepatic cells. Another possible explanation for the preserved liver morphology is the indirect effect of improved intestinal health. A well-developed intestinal barrier reduces the translocation of harmful bacteria and endotoxins into the bloodstream, thereby lowering hepatic inflammatory burden. Probiotics that stabilize gut microbiota and enhance mucosal integrity are therefore often associated with improved liver condition in fish [81,84]. In the present study, the increase in beneficial bacterial populations and the improved intestinal structure likely contributed to maintaining hepatic homeostasis.

### 4.5. Gene Expression Analysis

Dietary *Lysinibacillus* significantly influenced the expression of immune-related genes in the intestine, indicating an immunomodulatory role. Transcript levels of *TLR2* and *MHC II* increased progressively with higher probiotic doses. *TLR2* is a pattern-recognition receptor that detects Gram-positive bacterial ligands [88], so its upregulation likely reflects the immune system’s recognition of *Lysinibacillus* cell wall components and a resultant priming of innate immunity. Elevated *MHC II* suggests enhanced antigen presentation capacity, potentially improving the host’s ability to activate specific immune responses (e.g., priming T cells against pathogens) [89,90,91]. In contrast, proinflammatory cytokine genes (*IL-1β*, *TNF-α*, *IFN-β*) were not induced by a probiotic diet and *TNF-α* was slightly downregulated at the highest dose. The increase in pathogen-recognition and antigen presentation markers, without a surge in inflammatory cytokines, implies that *Lysinibacillus* sp. PWR01 triggered a measured, non-pathological immune stimulation. This immunomodulation is desirable as it suggests the probiotic can bolster immune readiness while avoiding chronic inflammation that could harm growth [14,92]. These findings mirror observations in other probiotic studies. Rwezawula, et al. [93] reported that tilapia fed *L. fusiformis* showed modulation of immune gene expression; for example, proinflammatory markers (*IL-1β*, *IL-6*, *CXCL8*) were moderated and the complement gene *C3* was upregulated, indicative of enhanced mucosal immunity coupled with reduced systemic inflammation. Similarly, in our study the lack of *IL-1β*/*TNF* surge and the improvement in survival post-infection point to an immune system that is both activated and balanced. It is noteworthy that not all probiotics behave identically in this regard. For example, a recent trial in common carp using a halophilic *Bacillaceae* probiotic (*Virgibacillus salarius*) found significant upregulation of *IL-1β* and *TNF-α* in probiotic-fed fish, which the authors associated with an acute immune activation contributing to pathogen defense [94]. By contrast, the *Lysinibacillus* in our study appears to have achieved protective effects without eliciting a strong inflammatory cytokine response. This difference could be due to species-specific immune dynamics or varying properties of the probiotic strains (e.g., differences in cell wall components such as lipoteichoic acids that drive cytokine responses). Mechanistically, probiotics are thought to act as immune modulators by interacting with gut-associated lymphoid tissue as they can stimulate toll-like receptors on intestinal immune cells, leading to enhanced phagocytic activity, local antibody production, and release of regulatory cytokines [95,96,97]. Many spore-forming bacilli also produce metabolites like short-chain fatty acids, peptidoglycans that have systemic immunological effects, including anti-inflammatory properties [98]. In practical terms, the upregulation of *TLR2* and *MHC II* observed here may translate to faster recognition of invading bacteria and more robust activation of adaptive immunity during infection, thereby improving disease outcomes. Indeed, the positive correlations noted between these immune genes and growth performance metrics in our multivariate analysis suggest a linkage between immune competency and overall vigor of the fish.

### 4.6. Disease Resistance Against A. hydrophila

The ultimate test of the probiotic’s efficacy was the challenge with the pathogen *A. hydrophila*. Fish that had been fed *Lysinibacillus*-supplemented diets showed markedly higher survival following infection compared to the positive control (infected but no probiotic). By day 14 post-challenge, the highest dose yielded the highest survival, and both 10^5^ and 10^6^ groups had significantly better survival than the control. These results provide strong evidence that encapsulated *Lysinibacillus* enhanced the fish’s resistance to bacterial infection. This finding is in agreement with multiple recent studies documenting probiotic-mediated disease protection in aquaculture. Qiu, et al. [30] similarly observed that zebrafish fed *L. sphaericus* (particularly at 10^7^ CFU/mL) had significantly improved survival when challenged with *A. hydrophila*, attributable to the probiotic’s immune-boosting effects. In Nile tilapia, *Bacillus* and *Lysinibacillus* supplementation has conferred protection against various pathogens; for instance, probiotic-fed tilapia challenged with *Providencia* experienced higher survival and reduced infection signs relative to controls [93]. Mechanistically, the enhanced survival can be explained by a combination of the probiotic’s direct antagonism of the pathogen and the primed host immunity discussed above. *Lysinibacillus* sp. PWR01 may produce antimicrobial compounds or bacteriocins that inhibit *A. hydrophila* either in the gut or systemically. Additionally, an improved gut microbiota (with more LAB and overall bacteria) could outcompete or inhibit *A. hydrophila* colonization. The significant positive correlations we observed between the abundance of gut microbiota and immune markers (*TLR2*, *MHC II*) suggest that a probiotic-enriched microbiome was associated with an immune status that is better prepared to fight infections. This is supported by other work showing that probiotics can suppress pathogen virulence factors and reduce pathogen load in hosts [28,99,100]. Furthermore, immune-enhancing effects such as higher lysozyme activity, complement activity, and Ig levels have been documented in probiotic-treated fish, contributing to more effective clearance of invading bacteria [101]. It is also worth noting that the survival curves in our study plateaued after day 6–7 in all probiotic groups, indicating the acute phase of infection was better managed in those fish, after which mortality ceased. In contrast, the positive control continued to suffer losses up to day 7 and ended at a much lower survival. This pattern is emblematic of an effective immunoprophylactic intervention, where the probiotic-treated hosts quickly control the infection. The outcome is highly relevant for aquaculture, as *Aeromonas* species cause septicemia and ulcers in many freshwater fish, and a 30% improvement in survival as seen with the high-dose *Lysinibacillus* could translate to significantly reduced economic losses. It also underscores that *Lysinibacillus* sp. PWR01 is a promising candidate for a biotherapeutic feed additive aimed at disease prevention.

### 4.7. Limitations and Future Directions

Beyond the applied outcomes, the study contributes novelty at both the strain-discovery and delivery-technology levels. *Lysinibacillus* sp. PWR01 was isolated from rubber latex nodules with evidence of chemically defended, enzyme and secondary metabolites. This rich microhabitat is increasingly recognized to harbor specialized endophytic communities [102]. Such latex-associated niches are plausibly selective for stress-tolerant, resilient bacteria, including endospore formers, because successful persistence requires coping with fluctuating osmotic pressure, oxidative stress, and antimicrobial plant compounds [103]. This ecological provenance therefore provides a biologically grounded rationale for the observed robustness of PWR01 during feed processing and gastrointestinal transit when combined with encapsulation. In parallel, recent aquaculture literature has begun to highlight *Lysinibacillus* spp. as emerging probiotics with measurable effects on host metabolism, intestinal function, and disease resistance [29,30,31], supporting the relevance of exploring non-traditional reservoirs for candidate strains.

Another innovation lies in positioning latex-derived *Lysinibacillus* as a complementary biocontrol option relative to conventional *Bacillus*-based probiotics. Although *Bacillus* probiotics remain effective and widely deployed, their antagonistic profiles can be strain-specific and sometimes targeted to a narrower set of pathogens. Recent studies demonstrate that individual *Bacillus* isolates may show broad-spectrum activity against *A. hydrophila* and other aquatic pathogens, but these effects are not universal across all commercial strains [104]. In this context, the present work provides academic value by demonstrating that an encapsulated *Lysinibacillus* isolate can confer meaningful protection against *A. hydrophila* in vivo while also improving performance traits under a practical feeding duration. Moreover, the results complement recent findings in zebrafish showing that *L. sphaericus* supplementation can modulate gut physiology and improve resistance to *A. hydrophila* [30], reinforcing the plausibility of *Lysinibacillus* as a disease-mitigating genus in aquatic hosts.

Despite these promising outcomes, several limitations should be acknowledged. First, the current study did not identify the fate of the encapsulated *Lysinibacillus* in the gut like levels of probiotic recovery from intestine or whether it became a persistent colonizer. Future work could use molecular tracing or selective plating to confirm that *Lysinibacillus* sp. PWR01 successfully colonizes or at least transiently proliferates in the fish gut at effective levels. Second, while key immune transcripts were measured, we did not assess protein-level or functional immune parameters such as phagocytic activity, lysozyme, or specific antibody levels. Including those in future studies would provide a more comprehensive picture of immunological changes. Third, the antioxidant findings (discrepancy between SOD and ABTS results) warrant deeper investigation into oxidative stress pathways; additional markers like catalase, glutathione, or expression of antioxidant genes could clarify how the probiotic influences redox homeostasis. Another limitation is that our trial was conducted in controlled laboratory conditions; performance and health benefits might differ in commercial pond or cage environments where fish are exposed to a more complex array of stressors and pathogens. Field trials will be important to validate the consistency of these benefits in real-world farming scenarios. We also note that the optimal dose observed here was in the 10^5^–10^6^ CFU/g range for feed which remains to be tested whether even higher doses (10^7^–10^9^) yield further gains or simply plateau, as some evidence suggests a saturation effect beyond a certain probiotic level. Additionally, while *Lysinibacillus* sp. PWR01 was effective alone, combining it with other beneficial microbes or prebiotics (synbiotic approaches) could be explored to amplify effects. There is growing interest in multi-strain probiotic formulations that target different aspects of host physiology. For instance, a mix of *Lysinibacillus* for immunity and a yeast or *Lactobacillus* for gut health. Finally, long-term safety and any potential trade-offs should be assessed. Thus far, no adverse effects were observed, consistent with other studies where *Lysinibacillus* strains have been deemed safe at high doses in fish, but thorough evaluations will strengthen the case for regulatory approval and industry adoption.

## 5. Conclusions

Dietary supplementation with encapsulated *Lysinibacillus* sp. PWR01 improved growth performance, antioxidant capacity, intestinal microbiota composition, immune gene expression, and disease resistance in a dose-dependent manner. Among the tested concentrations, 10^5^–10^6^ CFU/g emerged as the optimal supplementation range, achieving maximal growth enhancement and feed efficiency while significantly strengthening host antioxidant defense and immune responsiveness. Histological examination indicated that fish in encapsulated groups improved intestinal morphology by increasing villus height and width while maintaining normal muscular layer thickness and liver architecture, supporting enhanced intestinal structural integrity without observable tissue damage. Multivariate analyses showed strong positive associations between growth indices, beneficial microbiota, and immune activation, and clearly discriminated encapsulation treatments. These enhancements significantly improved survival rates following an *A. hydrophila* challenge, particularly at concentrations of 10^5^–10^6^ CFU/g. This finding underscores the practical application of encapsulated *Lysinibacillus* sp. PWR01 as a functional feed additive to enhance disease resistance in aquaculture. Collectively, this study provides the first evidence that encapsulated *Lysinibacillus* sp. PWR01 can serve as an effective probiotic in Nile tilapia, offering a practical strategy for optimizing probiotic dosage and delivery technology in aquaculture systems. The findings contribute mechanistic insight into microbiota-immune-growth interactions and support the application of encapsulated spore-forming probiotics as sustainable alternatives to antibiotic use in intensive fish farming.

## Figures and Tables

**Table 1:** Primer sequences used for quantitative PCR analysis in this study.

Gene	Primer Direction	Sequence (5′→3′)	Accession Number
*β-actin*	Forward	GGCATCACACCTTCTACAACGA	KJ126772.1
	Reverse	ACGCTCTGTCAGGATCTTCA	
*TNF-α*	Forward	GAACACTGGCGACAAAACAGA	AY428948
	Reverse	TTGAGTCGCTGCCTTCTAGA	
*IL-1β*	Forward	AGCTCCATGCAGTGATGCTG	XM_019365841.2
	Reverse	TGTTTTTATCCGTCACCTCCTCC	
*IFN-β*	Forward	CCATGAGCAGGAGAAACTTCT	—
	Reverse	GTGAGCTCGCCTCTTCGTACA	
*TLR2*	Forward	AAAAGCATAGATGAGTTCCACATCC	JQ809459.1
	Reverse	GTAAGACAAGGCATCACAAACACC	
*MHC II*	Forward	ATCACTGACTGGGATCCCTCCTC	NM_001279562.1
	Reverse	CTCGAGCCTTCCTCCTGTAGTAGAT	

**Table 2:** Growth performance parameters of Nile tilapia (*Oreochromis niloticus*) fed diets containing different *Lysinibacillus* sp. PWR01 encapsulation concentrations (0, 10^4^, 10^5^, and 10^6^ CFU/g) measured at weeks 4 and 8.

Parameter	Encapsulation Concentration (CFU/g)			
	0 (Control)	10^4^	10^5^	10^6^
Initial weight (g)	10.82 ± 0.08	10.80 ± 0.09	10.77 ± 0.03	10.83 ± 0.10
Week 4				
Survival rate (%)	100.00 ± 0.00	100.00 ± 0.00	100.00 ± 0.00	100.00 ± 0.00
Final weight (g)	26.00 ± 1.09	26.25 ± 0.65	27.63 ± 0.54	27.33 ± 0.73
Weight gain (g)	15.18 ± 1.08	15.45 ± 0.73	16.87 ± 0.55	16.50 ± 0.83
Percent weight gain (%)	137.83 ± 9.81	143.09 ± 7.74	156.66 ± 5.31	152.36 ± 9.04
Specific growth rate (%/day)	2.92 ± 0.11	2.96 ± 0.11	3.14 ± 0.07	3.08 ± 0.12
Daily weight gain (g/day)	0.54 ± 0.07	0.55 ± 0.03	0.60 ± 0.03	0.59 ± 0.03
FCR	0.84 ± 0.05	0.83 ± 0.03	0.84 ± 0.02	0.83 ± 0.01
Week 8				
Survival rate (%)	100.00 ± 0.00	100.00 ± 0.00	100.00 ± 0.00	100.00 ± 0.00
Final weight (g)	46.85 ± 1.08 ^b^	47.03 ± 2.42 ^b^	49.45 ± 1.08 ^ab^	51.48 ± 0.54 ^a^
Weight gain (g)	36.03 ± 1.13 ^b^	36.23 ± 2.37 ^b^	38.68 ± 1.08 ^ab^	40.65 ± 0.44 ^a^
Percent weight gain (%)	333.17 ± 12.20 ^c^	335.45 ± 20.68 ^bc^	359.29 ± 10.00 ^ab^	375.23 ± 1.86 ^a^
Percentage specific growth rate (%/day)	4.89 ± 0.09 ^b^	4.90 ± 0.16 ^b^	5.08 ± 0.07 ^ab^	5.20 ± 0.01 ^a^
Daily weight gain (g/day)	0.64 ± 0.02 ^b^	0.65 ± 0.04 ^b^	0.69 ± 0.02 ^ab^	0.73 ± 0.01 ^a^
FCR	1.52 ± 0.17 ^b^	1.37 ± 0.07 ^b^	1.24 ± 0.02 ^a^	1.31 ± 0.04 ^a^

Data are presented as the mean ± standard deviation (SD) (*n* = 3 per treatment). Within the same row and sampling time (week 4 or week 8), mean values followed by distinct superscript lowercase letters (a, b, c) indicate statistically significant differences among encapsulation concentrations. These differences were determined using one-way ANOVA followed by Duncan’s multiple range test (*p* < 0.05). Means sharing at least one common superscript letter are not significantly different (*p* > 0.05).

**Table 3:** Pairwise comparisons of post-challenge survival among treatments using the chi-square (χ^2^) test following *A. hydrophila* infection.

Treatment A	Treatment B	χ^2^ (Chi-Square)	*p*-Value
Control (+)	Control (−)	50.132	<0.001
Control (+)	Encapsulation 10^4^ CFU/g	1.454	0.228
Control (+)	Encapsulation 10^5^ CFU/g	4.226	0.040
Control (+)	Encapsulation 10^6^ CFU/g	5.719	0.017
Control (−)	Encapsulation 10^4^ CFU/g	34.907	<0.001
Control (−)	Encapsulation 10^5^ CFU/g	26.893	<0.001
Control (−)	Encapsulation 10^6^ CFU/g	23.929	<0.001
Encapsulation 10^4^ CFU/g	Encapsulation 10^5^ CFU/g	0.714	0.398
Encapsulation 10^4^ CFU/g	Encapsulation 10^6^ CFU/g	1.398	0.237
Encapsulation 10^5^ CFU/g	Encapsulation 10^6^ CFU/g	0.118	0.732

## Data Availability

The original contributions presented in this study are included in the article. The original RNA sequencing data presented in the study are openly available in the National Center for Biotechnology Information (NCBI) database (Accession No. PX415241.1). Further inquiries can be directed to the corresponding author(s).
